# “The Environment is Everything That Isn't Me”: Molecular Mechanisms and Evolutionary Dynamics of Insect Clocks in Variable Surroundings

**DOI:** 10.3389/fphys.2015.00400

**Published:** 2016-01-12

**Authors:** Gustavo B. S. Rivas, Luiz G. S. da R. Bauzer, Antonio C. A. Meireles-Filho

**Affiliations:** ^1^Laboratório de Biologia Molecular de Insetos, Instituto Oswaldo Cruz, Fundação Oswaldo CruzRio de Janeiro, Brazil; ^2^Laboratório de Fisiologia e Controle de Artrópodes Vetores, Instituto Oswaldo Cruz, Fundação Oswaldo CruzRio de Janeiro, Brazil; ^3^Centro de Desenvolvimento Tecnológico em Saúde, Fundação Oswaldo CruzRio de Janeiro, Brazil; ^4^Laboratory of Systems Biology and Genetics, Institute of Bioengineering, École Polytechnique Fédérale de LausanneLausanne, Switzerland; ^5^Swiss Institute of BioinformaticsLausanne, Switzerland

**Keywords:** circadian, photoperiod, entrainment, latitude, environment, cline, evolution

## Abstract

Circadian rhythms are oscillations in behavior, metabolism and physiology that have a period close to 24 h. These rhythms are controlled by an internal pacemaker that evolved under strong selective pressures imposed by environmental cyclical changes, mainly of light and temperature. The molecular nature of the circadian pacemaker was extensively studied in a number of organisms under controlled laboratory conditions. But although these studies were fundamental to our understanding of the circadian clock, most of the environmental conditions used resembled rather crudely the relatively constant situation at lower latitudes. At higher latitudes light-dark and temperature cycles vary considerably across different seasons, with summers having long and hot days and winters short and cold ones. Considering these differences and other external cues, such as moonlight, recent studies in more natural and semi-natural situations revealed unexpected features at both molecular and behavioral levels, highlighting the dramatic influence of multiple environmental variables in the molecular clockwork. This emphasizes the importance of studying the circadian clock in the wild, where seasonal environmental changes fine-tune the underlying circadian mechanism, affecting population dynamics and impacting the geographical variation in clock genes. Indeed, latitudinal clines in clock gene frequencies suggest that natural selection and demography shape the circadian clock over wide geographical ranges. In this review we will discuss the recent advances in understanding the molecular underpinnings of the circadian clock, how it resonates with the surrounding variables (both in the laboratory and in semi-natural conditions) and its impact on population dynamics and evolution. In addition, we will elaborate on how next-generation sequencing technologies will complement classical reductionist approaches by identifying causal variants in natural populations that will link genetic variation to circadian phenotypes, illuminating how the circadian clock functions in the real world.

## Introduction

The environment is the biotic and abiotic surroundings of an organism and one of the most powerful driving forces behind evolution. Among its many facets, the daily environmental cycles of light and temperature generated by Earth's rotation around its own axis are essential ecological factors shaping a variety of adaptations in living beings. In order to harmoniously follow these periodic changes, most organisms, ranging from bacteria to humans, have evolved a genetic multi-oscillatory timekeeping mechanism known as circadian clock (from the Latin *circa* meaning “about” and *dies* meaning “day”; Pittendrigh, 1993). This clock runs with a period of about 24 h and temporally organizes organismal physiology, metabolism and behavior to resonate with Earth geophysical cycles.

Because Earth axis is tilted approximately 23.4° there are, besides circadian fluctuations of environmental conditions, seasonal changes in day length (photoperiod) and temperature throughout the year (Daan, 2010). While nearly constant close to the tropics, these seasonal variations are striking at higher latitudes and constitute a strong selective force for animals and plants. Being poikilothermic, insects face daily environmental changes as a critical challenge for their survival and reproductive success, which ultimately shaped their adaptation to practically all environments on the planet. As with the circadian clock, this strong selective pressure on virtually all insects shaped a photoperiodic mechanism that interprets changes in photoperiod and temperature over the year in order to modulate metabolism and behavior to enhance survival in adverse conditions (Koštál, 2011).

While the circadian clock in insects modulates daily rhythms of activity/rest, eclosion, mating and feeding (Clements, 1999; Saunders, 2002), the most prominent outcome of the photoperiodic mechanism is the diapause, a programmed halt of development associated with changes in metabolism, physiology, and behavior (Schiesari and O'Connor, 2013). But although the interplay between the circadian and photoperiodic clocks has been widely reported in many insect species (reviewed in Bradshaw and Holzapfel, 2010; Saunders, 2010; Koštál, 2011; Saunders, 2013; Dolezel, 2015), it has been difficult to delineate their limits and overlap for mainly two reasons. The first is historical: while the molecular study of the circadian clock has immensely benefited from the genetic tools available for the insect model organism *Drosophila melanogaster* (see below), the lack of a pronounced seasonal response in this species prevented its use for photoperiodic clock studies (Tauber and Kyriacou, 2001). The second is related to the environment: although seminal behavioral observations in nature were pivotal to disclose general patterns of daily and seasonal activity in many insect species (Clements, 1999; Saunders, 2002), the influence of multiple environmental cues that varied in a seasonal and regional manner such as light, temperature, humidity, moonlight, social factors and even food availability, acted as strong confounding factors that made the characterization of circadian and photoperiodic clocks in nature a difficult task.

To circumvent the high complexity of the natural environment, experiments in the late 1950's/early 60's moved toward the lab, where environmental variables could be strictly controlled and tested separately (e.g., Pittendrigh, 1954). In addition, in controlled laboratory conditions it was possible to test the truly endogenous rhythm of a species by removing virtually all environmental conditions, i.e., placing individuals in constant darkness and temperature. This permitted the characterization of the endogenous circadian clock basic parameters (period and the phase), as well as its correlation with cyclic changes of behavior and physiology. This move, allied to powerful genetic screens in *D. melanogaster* that started in the late 60′s has set this species as the most important model in the study of circadian rhythms. Since then, *Drosophila* has contributed immensely to our knowledge on the molecular underpinnings of the circadian clock, and the role of light and temperature as synchronizing factors (also termed *zeitgebers*—“time giver” in German) to harmonize the clock with the changing environment.

Most of the seminal experiments in *Drosophila* used an artificial regime that roughly resembled daily changes of light and temperature at lower latitudes (12 h of light followed by 12 h of darkness in constant temperatures). However, more recent studies simulating conditions at higher latitudes revealed unappreciated clock plasticity at both molecular and cellular levels (Majercak et al., 1999; Rieger et al., 2003; Shafer et al., 2004). When more recently the experimental system was set outside the lab and the impact of all environmental variables were synchronously tested, the fruit fly behavior showed to be dramatically different from what was reported in controlled laboratory studies (Vanin et al., 2012; Das et al., 2015; Green et al., 2015). These late studies have highlighted that although many remarkable advances in understanding the molecular features of circadian clocks have been achieved inside the lab, only in the dynamic natural environment will the circadian and photoperiodic clocks be completely understood.

Crucially, only in the natural environment the adaptive value of circadian rhythms can be tested. Although traits that are environmentally sensitive are not necessarily adaptive, latitudinal clines in circadian genotypes and phenotypes in *Drosophila* have suggested adaptive circadian aspects in populations from different environments, suggesting the action of natural selection (Costa et al., 1992; Sawyer et al., 2006; Tauber et al., 2007).

In this review we discuss past and more recent studies on the role of the circadian clock in the natural environment and its impact on insect population dynamics, especially in *Drosophila*. We describe relevant findings in laboratory conditions related to the clock molecular machinery and the importance of external cues in clock synchronization and temporal niche determination. We discuss how setting up experiments outside the lab has changed dramatically our understanding of how seasonal environmental changes fine-tune the underlying circadian mechanism, which affects population dynamics and geographical variation in clock genes. Indeed, here we describe the benefits of studying latitudinal clines and stress the importance of proper comparative studies between locations in different continents. Finally, we speculate how recent genomic techniques, integrated with molecular methods and evolutionary biology, will allow the identification of genomic regions that harbor evolutionarily significant changes associated with the circadian clock and its adaptation to specific environments.

## Insect activity in the field and adaptation to laboratory conditions

The literature on daily activity rhythms of insects in nature, especially among mosquitoes, is extremely extensive and covers a large number of species in many different locations (reviewed in Clements, 1999). For example, in a single study Lewis and Taylor used suction traps to describe the diurnal and nocturnal patterns of flight activity rhythms of 400 insect taxa at 46 different locations (Lewis and Taylor, 1965). This study reported differences in insects' day preferences and activity phases: while most species were diurnal and displayed unimodal behavior (one peak of activity during the day), some as *Drosophilidae* showed bimodal distribution of activity around dawn and dusk. Importantly, the authors noted that environmental factors shaped insect's activity: while light intensity influenced the duration of flight in day-flyers, temperature affected the overall abundance of trapped insects (Lewis and Taylor, 1965). Indeed, the overt rhythm of activity is controlled by the circadian clock, which is continuously modulated by the direct effects of environmental cycles in nature.

The first experimental paradigm to access exclusively endogenous-generated circadian rhythms was to analyze an overt rhythm in constant darkness (de Mairan, 1729), a fundamental concept used until the present days (Pittendrigh, 1993). Roubaud was the first to apply this experimental design to an insect, and observed that *Anopheles maculipennis* was active only in the first 2 h of the night. These flight activity rhythms persisted in constant darkness and free-run under endogenous control with a period of 20–22 h (Roubaud, 1918). Hence, many studies used a system that recorded acoustic signals generated by individual flight activity to describe inter-specific period lengths, effects of light, temperature, insemination and blood feeding in many mosquito species (Jones, 1964; Jones et al., 1967; Clements, 1999). Later, more sophisticated systems based on infrared beam interruptions were developed for *Drosophila* (e.g., Tomioka et al., 1997; Helfrich-Förster, 1998), and commercial solutions made available (http://www.trikinetics.com). The Trikinetics system has been widely used in *D. melanogaster* (e.g., Glaser and Stanewsky, 2005; Sehadova et al., 2009; Kumar et al., 2014) and adapted for non-drosophilid insects such as mosquitoes and sandflies (Meireles-Filho et al., 2006; Rivas et al., 2008; Gentile et al., 2009; Rund et al., 2012). These experimental procedures were important to reproduce the first reports in the wild and to set the basis for tackling the circadian clock from the molecular perspective in controlled laboratory conditions. As the molecular description of circadian clock in most insects is not as well-described as in *Drosophila*, in the next section we will focus our attention on the fly work, while we refer the readers to recent reviews in other species (Bloch, 2010; Reppert et al., 2010; Tomioka et al., 2012; Meireles-Filho and Kyriacou, 2013; Tomioka and Matsumoto, 2015).

## The molecular circadian clock in *Drosophila melanogaster*

The seminal work of Konopka and Benzer was crucial to identify the genetic basis of circadian clock. They screened chemically mutagenized *D. melanogaster* flies searching for individuals with different circadian phenotypes in constant darkness, and isolated mutations that turned locomotor activity rhythms shorter, longer or totally disrupted compared to wild type flies. These three mutations mapped on same locus, which was named *period* (*per*) and constituted the first behavioral gene identified using mutagenesis (Konopka and Benzer, 1971). Later, after the *per* gene was cloned and characterized, its expression was shown to fluctuate in a circadian manner at the mRNA and protein levels in fly heads (Reddy et al., 1984; Jackson et al., 1986; Hardin et al., 1990).

**Figure 1:**
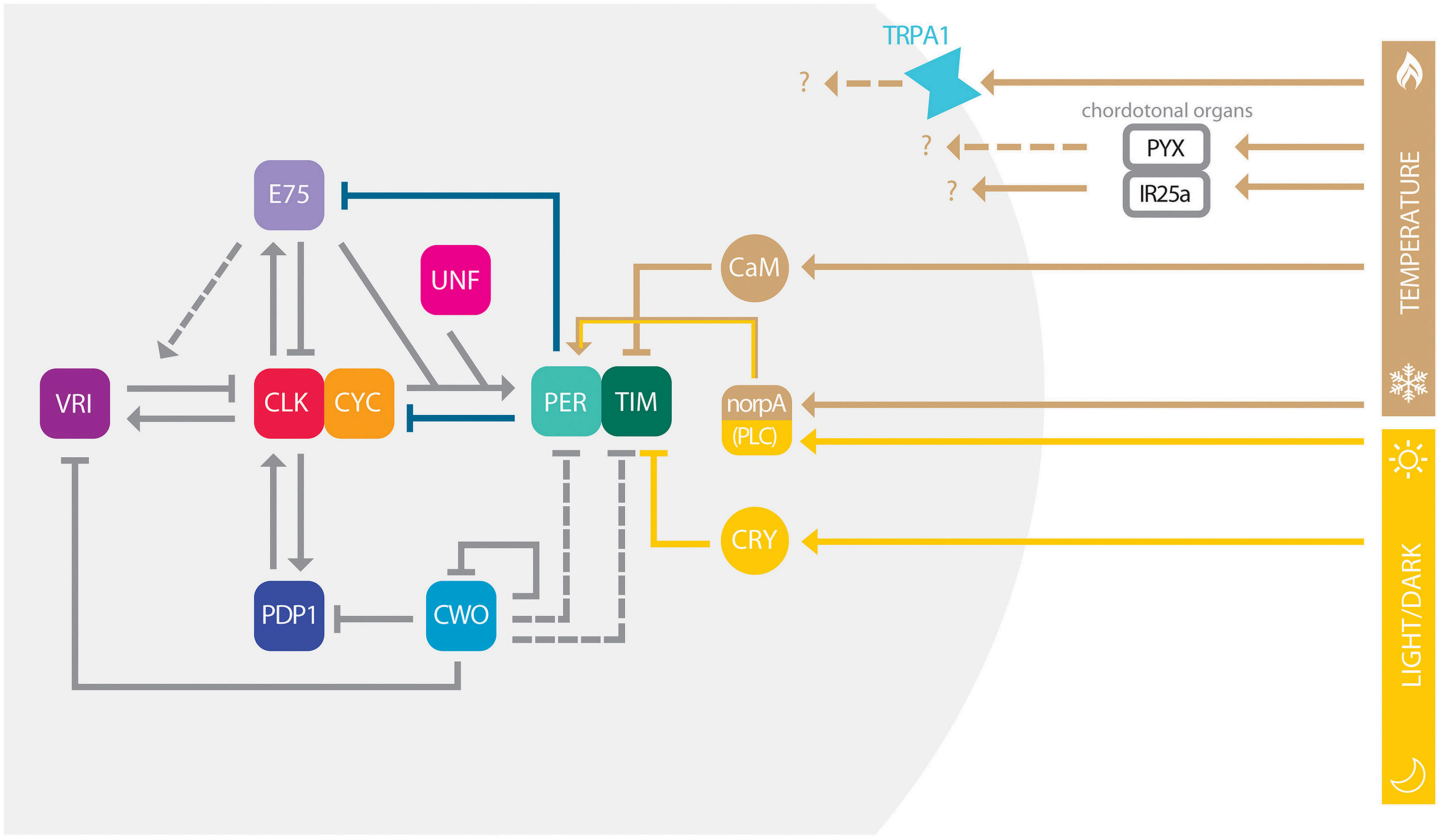
**Conceptual model of circadian clock in ***Drosophila*****. The model depicts multiple interlocked loops, which are centrally governed by the heterodimer CLK-CYC. For simplicity, we do not differ genes from regulatory sequences and proteins, in each case (e.g., PER) these are represented by their names and colored boxes. Arrows indicate positive/enhanced regulation, while blunted ends indicate negative/inhibitory regulation. Gray solid lines depict transcription regulation, while deep blue lines depict protein-protein interactions. Yellow lines show direction of light entrainment pathway, while brown lines depict temperature entrainment pathway. Dashed lines represent pathways that are plausible, but not fully described. While IR25a within the ChO is required for pacemaker synchronization, the roles of TRPA1 and PYX channels in temperature sensing remain to be elucidated. Temperature and light entrainment is also modulated by PLC encoded by *norpA*.

The circadian clock is based on interlocked transcriptional and translational feedback loops governed by transcription factors (TFs), which not only autonomously co-ordinate gene expression, but are also able to respond to changes in environmental conditions to maintain cellular homeostasis (reviewed in Hardin, 2011). In *Drosophila*, several transcriptional loops are interconnected by the TFs CLOCK (CLK) and CYCLE (CYC; Figure 1), which heterodimerize and bind to E-boxes sequences (*CACGTG*) in *cis*-regulatory regions close to the promoter of genes whose expression they control (Hao et al., 1997; Abruzzi et al., 2011; Meireles-Filho et al., 2014). In a first loop, CLK/CYC activates the expression of *per* and *timeless* (*tim*) (Allada et al., 1998; Darlington et al., 1998; Rutila et al., 1998), whose products accumulate in the cytoplasm during early night, heterodimerize and shuttle back into the nucleus to block CLK/CYC activity by forming an inactive multimeric complex PER/TIM/CLK/CYC (Lee et al., 1998, 1999; Bae et al., 2000; Yu et al., 2006). Without CLK/CYC mediated activation *per* and *tim* levels start to decrease, which leads to a decrease in PER and TIM levels that consequently releases CLK/CYC inhibition, allowing a new round of transcription to occur. In the second loop, CLK/CYC activates *vrille* (*vri*) and *Pdp1*ε expression (Figure 1; Cyran et al., 2003; Glossop et al., 2003). Although controlled by the same activator, VRI and PDP1 accumulate at different phases and compete with each other to bind on promoter region of *Clk*. Because VRI is a repressor and accumulates earlier than PDP1 (the activator), this lag of abundance promotes *Clk* circadian expression (Cyran et al., 2003; Glossop et al., 2003). In the third loop, CLK/CYC activates *clockwork orange gene* (*cwo*) expression (Figure 1), which codes a transcriptional repressor belonging to the basic helix-loop-helix (bHLH)/orange family of TFs. CWO also binds to E-box sequences and therefore competes with CLK/CYC binding to these regions, including its own promoter, as well as *vri* and *Pdp1* ones (Matsumoto et al., 2007). CWO negative regulation directly affects E-box-mediated transcription, contributing to general stability of the pacemaker (Kadener et al., 2007; Lim et al., 2007; Richier et al., 2008). Recently, two nuclear receptors were implicated in a new feedback loop: *unfulfilled* (*unf*) and *E75* (Figure 1). Although the precise molecular mechanism is still unclear, *unf* and *E75* knockdown flies are arrhythmic. Moreover they are both expressed in the circadian pacemaker neurons and collaborate to enhance CLK/CYC-mediated transcription of *per*, but curiously not *tim* (Beuchle et al., 2012; Jaumouillé et al., 2015). E75 alone binds and represses *Clk* expression and enhances VRI repression, suggesting that this nuclear receptor is important to modulate *Clk* regulation alone or together with VRI (Figure 1). In adittion, PER suppresses E75 activity, revealing a new role for PER as a de-repressor for *Clk* transcription (Kumar et al., 2014).

An emerging picture from the *Drosophila* circadian network is that as more factors are found to participate in the regulatory circuit, gene functions start to overlap (Figure 1), creating the robustness necessary for system stability across different environmental variables. Nevertheless, the stable circadian pacemaker is able to integrate multiple intra- and extra-cellular cues through TFs to produce specific patterns of gene expression necessary for physiological and behavioral responses. For example, the recently identified UNF and E75 belong to the class of nuclear receptors that have their activity modulated by small ligands. Although it is still not know which molecule binds UNF, E75 is known to bind heme and respond to gas (Reinking et al., 2005). E75 therefore might serve to couple the circadian clock with variable environmental CO levels or other ligands. Future mechanistic studies on clock TFs might elucidate how they sense signals from the environment to coordinate cellular responses of the circadian clock.

## Light and temperature entrainment

The endogenous clock is self-sustained but able to use external cues to adjust to a proper time in synchrony with the environment. The major agents of entrainment are light/dark cycles and temperature fluctuations. Insects use several structures and molecules to receive light and temperature inputs and pass it through to clock pacemaker.

In *D. melanogaster* a genome-wide expression analysis showed hundreds of genes responsive to light and temperature, some of them affected through photo and thermoreceptors within the circadian clock, while others were directly influenced by light or temperature independently (Boothroyd et al., 2007). Interestingly, temperature can affect a higher number of transcripts compared to light, which demonstrates how this environmental agent is important to entrainment. Indeed, low temperatures enhance the splicing of an intron in *per*, which advances phases of *per* mRNA and protein levels (Majercak et al., 1999). Consequently, flies in cold days are more diurnal than normal, which is an important seasonal adaptation during the winter adjusting the behavior to warmer temperatures. Similarly, an isoform of *tim*, termed *tim*^*cold*^ is dominant under low temperatures and in turn contributes to increase the overall *tim* transcript levels (Boothroyd et al., 2007). In fact, *tim* and *per* are both important for seasonal adaptation related to light and temperature due to polymorphic differences displayed among populations (see more details below).

The molecular mechanism of light/dark cycles entrainment is well-characterized in many species. In *Drosophila* this is mainly achieved by the flavoprotein *Cryptochrome* (CRY); *cry*^*b*^ mutant respond barely to light pulses and is still rhythmic under constant light, a condition that leads wild type flies to arrhythmicity (Stanewsky et al., 1998; Emery et al., 2000; Kistenpfennig et al., 2012). Light induces a conformational change that activates CRY, which then interacts with TIM and conduct it to proteasome degradation through the E3 ligase protein JETLAG (Suri et al., 1998; Yang et al., 1998; Ceriani et al., 1999; Naidoo et al., 1999; Lin et al., 2001; Busza et al., 2004; Koh et al., 2006; Peschel et al., 2006). In addition BRWD3, a substrate receptor for CRL4 (cullin 4 ring finger E3 ligase), has also been implicated in light induced CRY ubiquitination and degradation (Ozturk et al., 2013). As TIM degradation occurs only during daytime, TIM levels are restricted to the night. As PER is unstable without TIM, PER levels decrease in early morning and CLK/CYC inhibition is released, which is crucial to activate important output genes and dictate diurnal fly activity. In addition to CRY, light entrainment in flies is a result of multiple photoreceptors acting in synergy, since the entrainment by light is completely impaired when all external and internal photoreceptors are eliminated (Helfrich-Förster et al., 2001).

Not surprisingly, temperature entrainment depends on a functional clock since *per*^0^ mutants merely respond to temperature cycles (Wheeler et al., 1993; Yoshii et al., 2005). While in flies most organs independently entrain their local clocks with temperature cycles, the brain mostly relies on inputs from external thermal sensory structures such as the chordotonal organ (ChO; Sehadova et al., 2009; Simoni et al., 2014). It has been recently shown that the *pyrexia* (*pyx*) and the *Ionotropic Receptor 25a* (*IR25a*) genes are expressed in the ChO but exert different functions, the first being important to synchronization at lower temperatures and the other to perception of small temperature cycles (Wolfgang et al., 2013; Chen et al., 2015). In addition the *TrpA1* gene, which is expressed in clock and non-clock neurons in the *Drosophila* brain, might be important in the temperature-dependent regulation of afternoon siestas (Figure 1; Lee, 2013; Lee and Montell, 2013; Das et al., 2015; Green et al., 2015). At the molecular level temperature increases intracellular Ca^2+^ levels, which in turn triggers CALMODULIN(CaM)-mediated degradation of TIM by the Small Optic Lobe (SOL) protease (Tataroglu et al., 2015). As for the CRY-dependent light TIM depletion, temperature-mediated TIM degradation also resets the clock, showing that both light and thermal input pathways make use of TIM to synchronize the clock. Similarly *norpA*, which encodes phospholipase C, was also shown to be crucial not only for *Drosophila* phototransduction but also for temperature entrainment (Collins et al., 2004; Majercak et al., 2004; Glaser and Stanewsky, 2005), suggesting that light and temperature act not only independently but also in an integrated manner to compensate and reinforce the adaptation to a specific temporal niche. However, inside the laboratory, even when in combination, multiple *zeitgebers* are artificial and cannot reflect the complexity of the natural environment.

## Entrainment and non-circadian influence of environment in insect's rhythmicity

A pure expression of the endogenous circadian clock is generated in constant conditions. However, an animal's daily behavior is an interplay between the circadian pacemaker and immediate responses to environmental changes, which can be very different from what is observed inside the lab.

In some species there is a reasonable concordance in locomotor activity rhythms between the lab and nature. For example, the sandfly *Lutzomyia longipalpis* has a major peak of activity soon after lights off under 12:12 LD cycles (Meireles-Filho et al., 2006; Rivas et al., 2008) that persists in several other photoperiodic regimes (Rivas et al., 2014). This is in good agreement with the hourly abundance of eight wild species of sandflies captured in forested areas, which peak in abundance just after the sunset during summer (Guernaoui et al., 2006). Nevertheless, during autumn sandflies are scarcer at night and mostly peaked a little later (2 h after sunset), possibly influenced by differences in temperature and humidity levels between seasons (Guernaoui et al., 2006). Similarly, there are differences in overall activity levels depending on the lunar cycle: *Lutzomyia intermedia* and *Lutzomyia whitmani* females are more active during first, last quarters and full moon nights compared to moonless nights (Souza et al., 2005), a phenomenon also observed in crepuscular mosquitos (Provost, 1958; Bidlingmayer, 1964; Charlwood and Jones, 1980). Overall, these results suggest that even small variations in environmental conditions are able to influence overt activity rhythms.

Strikingly, there are cases where the “natural” behavior of a given species can be very different from what it is observed in the laboratory. For example, hamsters are crepuscular in the wild but nocturnal in laboratory (Gattermann et al., 2008), and mice that are strictly nocturnal in the laboratory, may be partially or completely diurnal in the field (Daan et al., 2011). As individuals in nature are daily influenced by a complex environmental scenario that varies seasonally and geographically, the temporal niche (diurnal, crepuscular, nocturnal) observed is a mixture of immediate responses to environmental changes and clock-controlled processes. Indeed, nocturnal animals switch the temporal niche to diurnal in conditions of low temperature or food restriction, which avoid energy expenditure in cold nights (Hut et al., 2013; van der Vinne et al., 2014, 2015). Being poikilothermic, insects exposed to different temperature levels can also change the onset activity and diurnal activity proportion (Lazzari, 1992; Majercak et al., 1999; Rivas et al., 2014) using the same adaptive strategy of homoeothermic organisms. In this sense, variable environmental cues can not only act on clock synchronization, but also produce a direct effect on behavior independently of it, a phenomenon termed “masking” (reviewed by Mrosovsky, 1999). Masking can inhibit or stimulate activity and disguise the clock-controlled phase of behavior. For example, *Drosophila malerkotliana* is diurnal under low but nocturnal under high light intensities (Sharma et al., 2012), a specific kind of masking called “paradoxical” (Mrosovsky, 1999), which is reinforced by the combined effects of bright light and high temperature (Sharma et al., 2012). Therefore, both masking and entrainment act in combination with the central clock to fine-tune overt daily rhythms of activity in nature.

In an attempt to elucidate how the molecular circadian clock operates in nature, numerous recent studies simulated different environmental conditions in the laboratory. One environmental component that is highly variable and known to affect the activity of insects, both through entrainment and masking, is light intensity. Circadian clocks are highly light-sensitive and can be influenced even by low irradiances that occur at dawn, dusk and moonlight (Rieger et al., 2007). In controlled laboratory conditions, *D. melanogaster* shows two peaks of locomotor activity around dawn and dusk (morning “M” and evening “E” peaks, respectively) and a period of less activity in the middle of day, a behavior that was termed the “siesta” (Hamblen-Coyle et al., 1992; Wheeler et al., 1993; Helfrich-Förster, 2000). In the laboratory fruit flies have a preference for low light intensities (between 5 and 10 lux) that allow the expression of different behavioral phenotypes, such as resting, grooming and feeding (Rieger et al., 2007). In this respect, exposure to artificial light similar to quarter-moonlight (0.03 lux) changes the phase of the endogenous clock, consequently shifting both peaks of locomotor activity into the night (Bachleitner et al., 2007). Importantly, clock mutants were also able to switch their temporal niche, suggesting that light bypassed the circadian clock to directly modulate fly activity patterns (Kempinger et al., 2009). Indeed, the compound eyes were recently shown to drive this masking effect in a clock-independent manner, both in moonlight perception (Kempinger et al., 2009; Schlichting et al., 2014) and in the timing of M and E peaks under natural-like conditions (Schlichting et al., 2015a). Therefore, under semi-natural conditions of moonlight or gradual light transitions at dawn and dusk (which mimics twilight), flies use mainly the visual system to determine locomotor activity peak times, where twilight has a more dominant role in fruit fly behavior than moonlight (Rieger et al., 2007; Schlichting et al., 2015b).

These somehow counter-intuitive results highlighted the difficulties associated in studying the combined effects of the environment in locomotor activity measurements inside the lab and encouraged more “natural” set ups.

## New perspectives and studies in semi-natural conditions

The simplicity of the locomotor automated system used by most fly labs allowed its adaptation to outdoor areas, once shaded places and rain protection were provided. Recently, Vanin et al. (2012) applied this set up in two different natural locations, Leicester, UK and Treviso, Italy and measured *D. melanogaster* locomotor activity through different seasons. Surprisingly, in contrast to what was expected from laboratory studies, flies were notably diurnal in the wild, with crepuscular activity not reaching 25% of the total amount recorded. In addition, disagreeing with the “siesta” observed in laboratory measurements at high temperature, the authors observed a major activity peak at midday during summer, which they termed the “A” (afternoon) peak. Importantly, unlike “A” and “E” peaks that are clock-modulated, only the *tim*^0^ mutant showed temperature independence in morning onset activity, suggesting that the “M” component is driven by twilight-dependent temperature cycles and not the circadian clock. Finally, temperature was more important for entrainment than light/dark cycles since locomotor behavior from flies in Leicester (UK) and Treviso (Italy) were quite similar, despite a considerable difference of daytime length between these regions in summer (Vanin et al., 2012).

As noted previously, given that environmental conditions are highly dependent on geographical location, comparisons between experiments performed in different regions should be made with caution, as it is virtually impossible to know all variables playing a role in nature. Nevertheless, following this study other groups used the same experimental procedure to measure fly locomotor activity outside the laboratory and reached similar observations. In Würzburg, Germany, Menegazzi and colleagues found only minor differences regarding the onset of the “E” peak in *per*^01^ mutant flies, which was delayed compared to wild-type flies in this study (Menegazzi et al., 2012) but anticipated in Vanin et al. (2012). Similarly, most of the previous findings were also observed in a region of South India, except that the authors of this study mistakenly suggested that the “A” peak was an artifact caused by the experimental procedure from Vanin et al. (2012) (De et al., 2013). Indeed, recent results confirmed the existence of the “A” peak with different methodological approaches (glass tubes used in TriKinetics monitors and in open-field arenas), in temperate and sub-tropical regions and even in De et al. (2013)'s own data, which overall suggests that the “A” peak is a clock-modulated escape response (Green et al., 2015). Although it is striking that these reports reached very similar conclusions despite of differences in latitude where these studies took place (52°N for Leicester, 45°N for Treviso, 49°N for Würzburg, and 12°N for South India), future work should aim to use even more natural conditions as in nature flies experience social, auditory, olfactory, and gustatory cues that influence their activity pattern. Altogether, these results show that even under different environmental conditions the circadian clock is able to operate in robust harmonic configurations.

Early findings in laboratory conditions had shown that even small differences of 2–3°C were able to phase-shift the *Drosophila* rhythmic locomotor behavior (Wheeler et al., 1993). In the wild, temperature cycles were especially important on the determination of the “A” component, which was shown to be dependent on the *TrpA1* channel expressed in *TrpA1*-expressing neurons other than the canonical clock ones (Das et al., 2015; Green et al., 2015). At the pacemaker level, *norpA* splices the 3' intron of the *per* mRNA transcript in a temperature-dependent manner, changing PER levels that consequently will advance or delay the circadian phase (Majercak et al., 1999, 2004; Collins et al., 2004). In semi-natural conditions, *per* mRNA cycling in fly heads is observed only in summer, while *tim* cycles robustly throughout the year (Montelli et al., 2015). Accordingly, PER but not TIM abundance follow seasonal changes in the fly brain (Menegazzi et al., 2013), confirming the importance of PER in interconnecting seasonal environmental changes to behavioral responses. Overall, these results highlight an interesting anatomical difference between light and temperature entrainment in flies: while light can activate CRY in a cell autonomous manner, temperature reaches the brain indirectly probably through several different channels expressed in peripheral organs.

Given the pivotal importance of temperature to dictate *Drosophila* daily activity in nature, these results should encourage further experiments upon the role and hierarchy of each thermoreceptor in regimes with conflicting gradual light and temperature cycles, especially among wild populations that show latitudinal clines.

## Genetic and phenotypic variation according to gradual environmental changes

Evolutionary studies indicate species as a group of organisms preserving their uniqueness that are often adapted to their local environment. The interaction between species and environment raises interesting questions. How can different sympatric organisms, such as plants and animals be so divergent if they are ruled by the commands of the same environmental conditions? In species showing broad range distribution pattern (covering large geographical transects such as latitude, altitude or longitude), how can allopatric individuals conserve their taxonomic identity if they are governed by the commands of different environmental conditions?

For many years, biologists have been trying to understand evolutionary forces influencing genetic variation within and among species (Lewontin, 1974; Hughes, 2007; Mitchell-Olds et al., 2007). It has been a long search to connect this genetic variation with variation in phenotypes and fitness within natural populations. One good possibility to tackle this problem is to study sample variation along geographic transects, which provides great benefit when compared with studies performed on patchy samples. Clines can be defined as predictable geographic gradients in a genotype or a phenotype that can be measurable (Endler, 1977) and can be replicable to a degree that variation sampled from patchy landscapes can not. The main benefits of studying clines include the possibility of parallel adaptation and the attenuation of some of the confounding effects of demography. A good example is that a cline along a coastal latitudinal transect can be potentially replicated on multiple continents, providing evidence of parallel adaptation. Indeed, one further classic way of differentiating between natural selection and drift in the generation of a latitudinal cline is to study the polymorphism in different continents (Oakeshott et al., 1981).

In *Drosophila* and other species, several studies were performed considering phenotypic, genetic, and genomic gradual change variation over the geographical range of the species distribution (reviewed by Kyriacou et al., 2008; Yerushalmi and Green, 2009; Costa and Stanewsky, 2013; Hut et al., 2013; Adrion et al., 2015). For example, *Drosophila littoralis* is a latitudinal widespread European species of the *Drosophila virilis* group that has ample genetic variation in photoperiodism (diapause in adults) and circadian rhythmicity (pupal eclosion), with adaptive latitudinal clines in both of them (Lankinen, 1986). Interestingly, it was shown that the adaptively variable gene *loci* are different for photoperiodism and the circadian clock, and that threonine-glycine repeat section of the *per locus* (a strong candidate for clock variability, see Clines in Clock Genes section) was not included in the polymorphism of *D. littoralis* clock genes (Lankinen and Forsman, 2006). Phenotypic differentiation along latitudinal transects has been shown for various traits in *D. melanogaster* and many patterns are recapitulated among continents (Adrion et al., 2015). Nevertheless, *D. melanogaster* shows a number of latitudinal clines in morphological characteristics such as body size (James et al., 1997) or in frequencies of various metabolic genes (Oakeshott et al., 1981, 1983a,b, 1984; Bubliy et al., 1999), suggesting that selection, in the face of considerable drift and migration, has already made its mark. Single molecular markers have also been used to elucidate clinal differentiation and spatial variation in allele frequencies, revealing variations that tracked the clines (reviewed in Kyriacou et al., 2008; Costa and Stanewsky, 2013; Hut et al., 2013). Those studies increased the scientific understanding of local adaptation and the characterization of selective forces determining the high diversity in phenotypes observed in nature.

**Figure 2:**
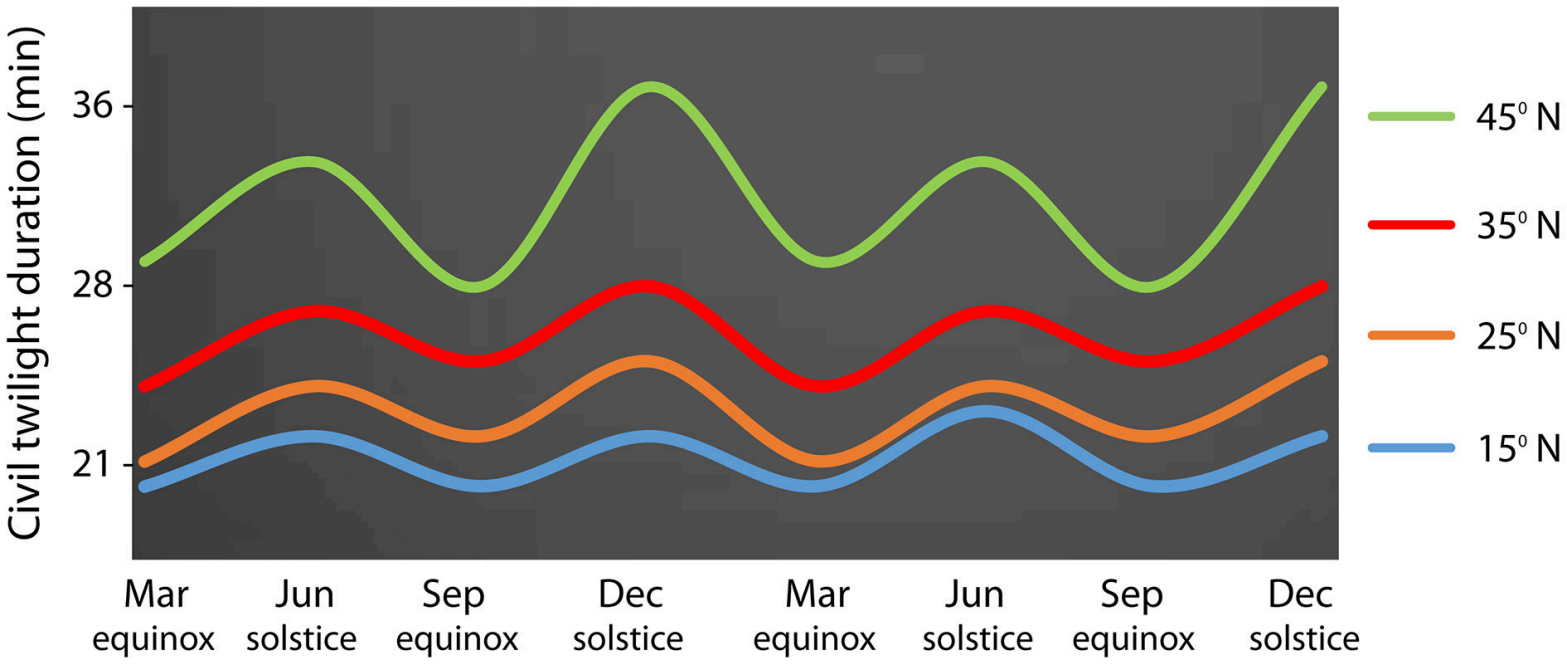
**Latitudinal variation in the duration of the civil twilight along two consecutive years** In the abscissa it is shown astronomical events (equinoxes and solstices). Latitude 15°N in blue, latitude 25°N in orange, latitude 35°N in red, latitude 45°N in green.

For a better understanding of latitudinal clines it is important to know the correlation between photoperiod and temperature (Hut et al., 2013). Contrary to temperature oscillations, the photoperiod parameter remains stable over the years, with the duration of the civil twilight being dependent on the latitude (Figure 2). Temperature is a parameter that is more affected by the distribution of landmass and sea currents over the globe, causing quantitative space and temporal differences. For instance, due to the ocean Gulf stream that affects climate conditions in Europe, Boston (USA, 42°N) is colder than London (51°N) in winter even though it is lower in latitude. Overall, while temperatures vary considerably over the years, the photoperiod remains stable. Organisms therefore use photoperiod as a proximate factor to tune the annual timing of physiology and behavior to changes in ultimate factors such as temperature (Hut et al., 2013). Studies at the molecular level performed for documentation of segregating molecular polymorphisms would benefit more if disentangling complex interacting traits in annual timing. Considering more predictable geographical parameters such as the photoperiod may simplify conclusions of molecular studies performed in latitudinal transects.

## Parallel cline studies performed between continents

**Figure 3:**
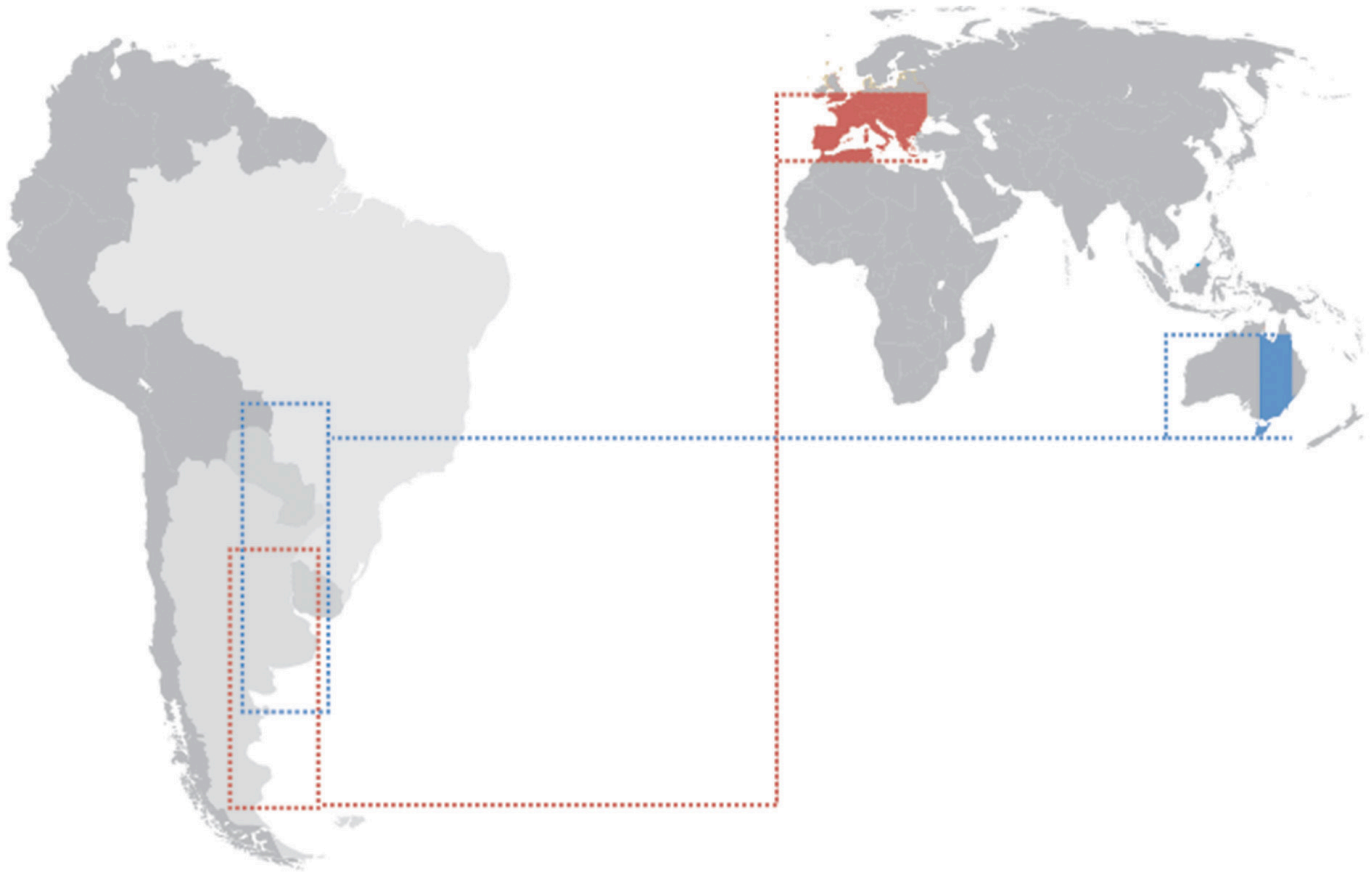
**Latitudinal clines in (Thr-Gly)_**20**_ frequencies studied in Europe (Costa et al., 1992 red) and Australia (Sawyer et al., 2006 blue) and how they would correlate if the considered latitudinal areas were transposed to South America**.

Evidence for parallel adaptation and identification of commonalities in the genes responding to cline selection have been performed among continents. For instance, North American and Australian eastern coastal clines have been heavily studied for both phenotypic and genetic variation (reviewed in Adrion et al., 2015). The North American cline has been sampled from southern Florida, USA (25°N) to Vermont and Maine USA (44°N). The Australian cline has been heavily sampled from northern Queensland (15°S) to Tasmania (43°S). There is high environmental variation along these clines. High latitude populations on both continents experience lower mean temperatures, greater variance in temperatures across season and reduced UV light exposure (Hoffmann and Weeks, 2007). The latitudinal range in Australia takes into account a large portion of tropical climate whereas the latitudinal cline range in the USA does not. This has to be considered with caution. For a proper parallel adaptation comparison the geographical ranges studied should take into account exactly the same latitudes. Because tropical populations below 25°N in the Americas have been under explored it is unknown if inclusion of theses populations would strengthen or lessen support for parallel adaptation among Australian and North American clines. Another problem is that the highest latitudes observed in Australia cover an island area (Tasmania) and this can bring specific demographic features such as restricted gene flow and inbreeding, which make populations living in such environments particularly prone to the effects of drift. The Southern hemisphere has less land and a wider ocean area compared to the Northern hemisphere. Also the land distribution in the South hemisphere hardly reaches high latitudes. South America is the only continent in the Southern hemisphere where a continuous land latitudinal cline transect can be determined, from low up to very high latitudes (Figure 3). Unfortunately almost nothing is known about the clinal genetics and history of South American populations. Although cline studies bring lots of advantages, one has to bear in mind that not all differentiation occurs in parallel among continents. There are cases where patterns are not repeatable among clines. For example, in contrast to the well-defined cline in North America, the incidence of diapause (Lee et al., 2011) and the number of ovarioles (Azevedo et al., 1996) in *Drosophila* display non-linear associations with latitude in eastern Australia.

## Clines in clock genes

Clinal variation in circadian genes has previously been identified in a variety of organisms such as salmonids (O'malley and Banks, 2008; O'malley et al., 2010), passerine birds (Johnsen et al., 2007) and plants (Chen et al., 2012; Keller et al., 2012), in addition to *D. melanogaster* (Costa et al., 1992; Sawyer et al., 2006; Tauber et al., 2007; Kyriacou et al., 2008). Moreover, properties of circadian rhythms in the same species living at different latitudes have been useful in examining the correlation of circadian rhythms with the environment, reaching the conclusion that having a circadian system that matches the oscillating environments is adaptive (Yerushalmi and Green, 2009).

One of the best known cline studies in circadian clock genes was performed with the *per* gene. Within PER there is a repetitive region composed of alternating dipeptides of threonine and glycine (Thr-Gly) residues (Yu et al., 1987). In *D. melanogaster*, the repeat is highly polymorphic, both in sequence and length (Costa et al., 1991). Repeat length variation in natural populations within Europe follows a latitudinal cline, so that high frequencies of the (Thr-Gly)_20_ and (Thr-Gly)_17_ alleles are found in Northern and Southern regions, respectively (Costa et al., 1992). Linkage disequilibrium pattern analysis in this region suggested that balancing selection may be operating and this would seem to fit in nicely with the clinal distribution (Rosato et al., 1997). Finally, taken together these two length alleles make up approximately 90% of the natural variation found in Europe. Variants carrying 14 (1%) and 23 (8%) Thr-Gly pairs and very rare variants with 18, 21, and 24 pairs (together counting for 1%) make up the rest. The temperature compensation of the clock differs among the Thr-Gly variants (Sawyer et al., 1997). The (Thr-Gly)_17_ variant has a 24 h cycle at higher temperature, but the period becomes shorter as the temperature is reduced. On the other hand, the (Thr-Gly)_20_ variant shows a period that is not sensitive to temperature change and is on average slightly shorter than 24 h, appearing to be better buffered against temperature swings (Sawyer et al., 1997).

**Figure 4:**
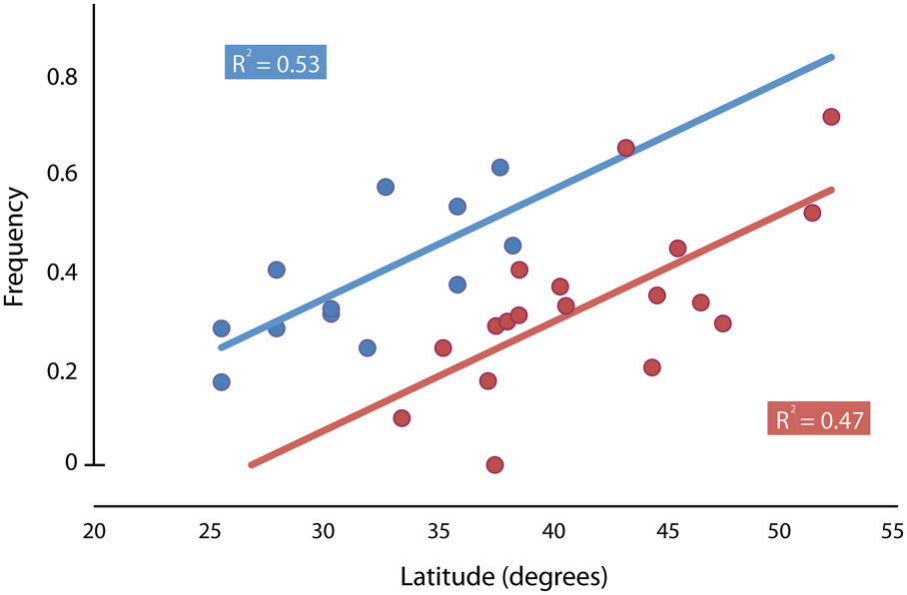
**Latitudinal variation of ***per*** (Thr-Gly)_**20**_ repeat length frequency in European (red dots—Costa et al., 1992) and Australian natural populations (blue dots—Sawyer et al., 2006)**. In order to adjust the analysis for the same latitudes, the tropical Australian samples (latitude < 25°) were not considered. Also, samples from the Australian state of Tasmania were not considered as conditions in an island may differ from conditions in the mainland, as well as the selective pressure acting on these flies.

A cline in Thr-Gly polymorphism in the *per* gene in Australian populations of *D. melanogaster* was reported to be weaker than the European one, and this observation was consistent with the view that natural selection was maintaining the polymorphism in both continents (Sawyer et al., 2006). This cline would be particularly interesting in view of the fact that *D. melanogaster* was introduced to Australia 100 years ago (Umina et al., 2005). However, the existence of clinal variation in the 17 or 20 repeats alleles in Australian *D. melanogaster* populations is a matter of debate (Weeks et al., 2006, 2007; Kyriacou et al., 2007). Nonetheless, it is important to point out that the (Thr-Gly)_20_ clinal distribution compared between Europe and Australia is in quite different latitude ranges (from 33.40 up to 52.10° of latitude in Europe and from 16.88 up to 42.88° of latitude in Australia). Maybe one of the reasons for the less steep (Thr-Gly)_20_ cline in Australia was due to the fact the data were not analyzed for the same latitude range when comparing the Thr-Gly distributions in Europe and Australia. Figure 3 shows the distribution cline ranges studied in Europe (Costa et al., 1992) and Australia (Sawyer et al., 2006), and how the distribution range would not overlap entirely if the collections were performed in South America, for instance. By plotting a distribution graph comparing the Thr-Gly frequencies between Europe and Australia and discarding the more tropical Australian data (latitude below 25°S), it can be observed that the frequencies of (Thr-Gly)_20_ alleles are as steep in Australia as the frequencies are in Europe (Figure 4). This fact confirms that in Australia the trend is toward a greater number of Thr-Gly alleles and higher heterozygosity in the tropical lower latitudes (Sawyer et al., 2006) raising the hypothesis that the relaxed thermal selection of the tropics is more forgiving than the European climate (Kyriacou et al., 2007). Indeed, this hypothesis is supported by studies performed in the Nahal Oren Canyon, Israel, which is a canyon separated by only 200 m where the two neighboring slopes have “tropical-like” or “European-like” microclimates. While the frequencies of (Thr-Gly)_20_ alleles were higher in the “European” slope, frequencies of (Thr-Gly)_17_ were higher on the “tropical” slope (Zamorzaeva et al., 2005). Overall these observations suggest that future studies should analyze more temperate latitudes in both continents in order to find parallel adaptations.

Studies on clines' polymorphisms in circadian genes do not always provide such clear confirmation of the adaptive value of circadian rhythms, as occurs in *Drosophila* with the two common alleles of the *tim* gene (*ls-tim* that expresses both long and short forms of TIM protein and *s-tim* that only gives the short form of the protein). *ls-tim* probably arose in southern Italy < 10,000 years ago and causes attenuated photosensitivity of the circadian clock (Sandrelli et al., 2007; Tauber et al., 2007). Unexpectedly, there is a significant latitudinal cline with more *ls-tim* in southern Europe and more *s-tim* in northern Europe. It was suggested that the distribution pattern of *tim* polymorphism reflects the adaptive advantage of the relatively new *ls-tim* allele spreading under directional selection from its place of origin (Tauber et al., 2007). This was confirmed by showing that the latitudinal cline was in fact a distance cline from the point of origin, and that the frequency of the new allele was proportional to the overland distance from southern Italy. The *ls-tim* allele can potentially confer several adaptive advantages in environments at high latitudes, both in winter and summer. First, as in winter the days become colder faster than they become shorter, the relative insensitivity of *ls-tim* to long summer photoperiods allow flies to undergo diapause in long cold days. Second, when flies are exposed to a quasi LL (constant light) in midsummer, a condition that leads them to arrhythmicity (Stanewsky et al., 1998), the relatively reduced circadian response to light allow their clock to keep running under such conditions (Sandrelli et al., 2007; Tauber et al., 2007). Besides *tim*, polymorphisms in the *couch potato* and *insulin-regulated PI3 kinase* genes have also been implicated in diapause expression (Williams et al., 2006; Cogni et al., 2014), and it will be interesting in the future to evaluate if these pathways converge or affect diapause independently. Generally, latitudinal variation seems to be a powerful tool to study circadian mechanisms and photoperiodic responses to reveal selective forces involved in daily and seasonal adaptation (Emerson et al., 2009; Hut and Beersma, 2011).

In the future one can rely on whole genome analyses of populations over a geographical transect to identify both large and fine scale clinal patterns. Regions of the genome that are strongly differentiated between samples can be identified revealing signatures of adaptation. Differentiated regions that overlap among multiple clines can provide evidence of parallel adaptation. It will be promising to check for highly differentiated sites in the genome between two samples in different continents and verify if they might cluster by environment than by continent, providing evidence for the role of the environment in shaping genotypic and also phenotypic traits.

## Discussion

Insects make use of the visual system, mechanosensory pathways and thermo and olfactory receptors to perceive the environment. These structures transduce multiple external cues into an environmentally sensitive gene network, the circadian clock. The circadian pacemaker processes external information within its network and regulates the expression of hundreds of genes, protein stability and protein abundance in order to daily synchronize many aspects of metabolism, physiology, and behavior with the environment.

Environmental oscillations are paramount ecological factors for generation of adaptive traits among several species. In this respect, adaptations to daytime or nighttime activity are dependent not only on direct and effective responsiveness to environmental changes (e.g., bright light intensity or midday high temperatures), but also on anticipating these changes. Thus, the proper resonation of the clock with the environment is of fundamental importance for an organism to adapt to its surroundings.

Clock genes in insects (and other multicelular eukaryotes) encode important factors that modulate hundreds of genes to drive cyclical changes in physiology and behavior. Correlations of circadian rhythms with the environment have been demonstrated in several species, providing mounting evidence that having a circadian system that matches the oscillating surrounds is adaptive (Yerushalmi and Green, 2009). In this sense, clock genes are excellent candidates for acting as speciation genes and can be used to unveil speciation in insects (e.g., Colot et al., 1988; Peixoto et al., 1993; Bauzer et al., 2002; Tauber et al., 2003; Araki et al., 2009; Rona et al., 2010). But as the circadian transcriptome of different insect species depicted that many genes display circadian expression (Ceriani et al., 2002; Ptitsyn et al., 2011; Rund et al., 2011), the exclusive choice of clock genes as candidates for evolutionary studies certainly would tell just part of the whole history. Moreover, some important adaptive traits based on findings in *Drosophila*, such as the molecular basis of temperature compensation, could not be extrapolated for other insect populations, such as mosquitoes and sand flies. For example, the Thr-Gly polymorphism in the *per* gene is pivotal for natural temperature compensation and adaptation of European populations *D. melanogaster* (Costa et al., 1992) but was not found in *per* of *D. littoralis* (Lankinen and Forsman, 2006) and in *per* orthologs from mosquitoes and sand flies (according to our search on vectorbase database—vectorbase.org). This suggests that other mechanisms for temperature compensation in these species might be encoded in other parts of genome and raises an important point that although conserved, the circadian mechanism has species-specific differences that demand dedicated unbiased approaches in order to reveal them.

In this sense, genomic technologies now provide unprecedented access to evolutionary genetics. Natural populations within and across continents that are expected to show considerable level of genetic diversity can be fully sequenced at reasonable costs and genotype-phenotype association studies conducted to access the genomic regions responsible for trait adaptation. Several quantitative genetics studies have been conducted in *D. melanogaster* (Pasyukova et al., 2000; Geiger-Thornsberry and Mackay, 2004; Nuzhdin et al., 2005; Remolina et al., 2012), and also in mosquitos such as *Aedes aegypti* (Bennett et al., 2005; Saavedra-Rodriguez et al., 2008; Reyes-Solis et al., 2014) and *Anopheles gambiae* (Zheng et al., 1997, 2003; Blandin et al., 2009). Comparative studies across different continents regarding latitudinal clines of morphological or circadian related traits will be pivotal to identify genes important for parallel adaptation of populations among continents. In this scenario, South America is quite interesting and in fact there are few reports describing the occurrence of latitudinal clines in some insect species in different regions of this continent (Bubliy et al., 1999; Gilchrist et al., 2004; Rosetti and Remis, 2013).

In this regard, recessive alleles complicate genetic association studies and hence the identification of causal genes in natural insect populations. Alternatively, inbred lines can be used for genetic analysis, particularly in conjunction with genome-wide markers or full genome sequences, to enhance the power for genetic association analyses. For example, in the past, inbred populations have been successfully used in evolutionary genetics studies (Falconer and Mackay, 1996; Lynch and Walsh, 1998). In *D. melanogaster*, the Drosophila Genetic Reference Panel (DGRP) was established as a set of 204 fully sequenced inbred lines (Mackay et al., 2012) that have been used in a number of studies from accessing the genetic basis of sexual gene expression differences (Massouras et al., 2012) to the gut immunocompetence in flies (Bou Sleiman et al., 2015). Therefore, the use of inbred lines in the clock-dependent adaptation studies has the potential to find unique genomic signatures important for *Drosophila* to adapt and predict natural variable surroundings.

It is tempting to recommend that functional genomics should follow the behavioral assays in natural conditions in *Drosophila* and other insect species. In addition to assaying different populations from the same species, multiple comparisons between evolutionary distant species in the same geographical locations might also reveal important phenotypic and genotypic aspects of adaptation related to convergent evolution, and thus provide a more generalist idea of adaptation of temporal niche in species through evolution.

## Author contributions

GBSR, LGSRB and ACAMF contributed to the conception, design, draft, and editing of this article.

### Conflict of interest statement

The authors declare that the research was conducted in the absence of any commercial or financial relationships that could be construed as a potential conflict of interest.
